# Molecular mechanics and dynamic simulations of well-known Kabuki syndrome-associated KDM6A variants reveal putative mechanisms of dysfunction

**DOI:** 10.1186/s13023-021-01692-w

**Published:** 2021-02-05

**Authors:** Young-In Chi, Timothy J. Stodola, Thiago M. De Assuncao, Elise N. Levrence, Swarnendu Tripathi, Nikita R. Dsouza, Angela J. Mathison, Donald G. Basel, Brian F. Volkman, Brian C. Smith, Gwen Lomberk, Michael T. Zimmermann, Raul Urrutia

**Affiliations:** 1 Genomic Sciences and Precision Medicine Center (GSPMC), Medical College of Wisconsin, Milwaukee, WI USA; 2 Bioinformatics Research and Development Laboratory, and Precision Medicine Simulation Unit, GSPMC, Medical College of Wisconsin, Milwaukee, WI USA; 3 Division of Research, Department of Surgery, Medical College of Wisconsin, Milwaukee, WI USA; 4 Division of Pediatric Genetics, Department of Pediatrics, Medical College of Wisconsin, Milwaukee, WI USA; 5 Department of Biochemistry, Medical College of Wisconsin, Milwaukee, WI USA; 6 Department of Pharmacology and Toxicology, Medical College of Wisconsin, Milwaukee, WI USA; 7 Clinical and Translational Sciences Institute, Medical College of Wisconsin, Milwaukee, WI USA

**Keywords:** KDM6A, Epigenetic regulators, Histone demethylase, Genomic variation, Kabuki syndrome, Mutational impact analysis, Protein structure, Molecular dynamics

## Abstract

**Background:**

Kabuki syndrome is a genetic disorder that affects several body systems and presents with variations in symptoms and severity. The syndrome is named for a common phenotype of faces resembling stage makeup used in a Japanese traditional theatrical art named *kabuki*. The most frequent cause of this syndrome is mutations in the H3K4 family of histone methyltransferases while a smaller percentage results from genetic alterations affecting the histone demethylase, KDM6A. Because of the rare presentation of the latter form of the disease, little is known about how missense changes in the KDM6A protein sequence impact protein function.

**Results:**

In this study, we use molecular mechanic and molecular dynamic simulations to enhance the annotation and mechanistic interpretation of the potential impact of eleven KDM6A missense variants found in Kabuki syndrome patients. These variants (N910S, D980V, S1025G, C1153R, C1153Y, P1195L, L1200F, Q1212R, Q1248R, R1255W, and R1351Q) are predicted to be pathogenic, likely pathogenic or of uncertain significance by sequence-based analysis. Here, we demonstrate, for the first time, that although Kabuki syndrome missense variants are found outside the functionally critical regions, they could affect overall function by significantly disrupting global and local conformation (C1153R, C1153Y, P1195L, L1200F, Q1212R, Q1248R, R1255W and R1351Q), chemical environment (C1153R, C1153Y, P1195L, L1200F, Q1212R, Q1248R, R1255W and R1351Q), and/or molecular dynamics of the catalytic domain (all variants). In addition, our approaches predict that many mutations, in particular C1153R, could allosterically disrupt the key enzymatic interactions of KDM6A.

**Conclusions:**

Our study demonstrates that the KDM6A Kabuki syndrome variants may impair histone demethylase function through various mechanisms that include altered protein integrity, local environment, molecular interactions and protein dynamics. Molecular dynamics simulations of the wild type and the variants are critical to gain a better understanding of molecular dysfunction. This type of comprehensive structure- and MD-based analyses should help develop improved impact scoring systems to interpret the damaging effects of variants in this protein and other related proteins as well as provide detailed mechanistic insight that is not currently predictable from sequence alone.

## Background

Our studies are focused on the use and optimization of structural bioinformatics methods for improving the interpretation of data derived from next generation sequencing in cases presenting with rare diseases [1–4]. Extensive studies in our laboratories have previously demonstrated that these approaches yield not only information on the damaging effects of likely pathogenic variants but also yield useful mechanistic information on their pathophysiological impact on human health. The current study focuses on the analyses of Kabuki-associated histone lysine-specific demethylase 6A, KDM6A, a well-known epigenomic regulator that functions as an eraser of methylated histone marks. KDM6A is encoded by an X-chromosome-linked gene and the protein selectively catalyzes demethylation of tri/di-methylated histone H3K27 residues [5, 6]. There are two main classes of histone demethylases: flavin adenine dinucleotide (FAD)-dependent amine oxidases (*e.g.* lysine-specific demethylase 1 [LSD1]), and Fe(II) and 2-oxoglutarate (2OG)-dependent hydroxylases, both of which operate by enzymatic oxidation/hydroxylation of a methyl group, followed by non-enzymatic loss of formaldehyde resulting in histone lysine demethylation [7]. The jumonji domain-containing histone demethylase KDM6A (also known as UTX), along with two other related members, KDM6B (JMJD3) and KDM6C (Y-chromosome-linked UTY), belong to the 2OG-dependent dioxygenase superfamily [7]. KDM6A regulates developmentally important genes as demonstrated both in cultured cells using KDM6A knockdown and in vivo using KDM6A-deficient mice [8–11]. Consequently, all pathogenic KDM6A variants, known to result in loss of function mutations, can disrupt cell, organ, and systems homeostasis, thus impacting human health. Indeed, pathogenic KDM6A variants are one cause of Kabuki syndrome, a rare multi-systemic disorder which presents in approximately one in every 32,000 newborns [12–14] and causes developmental defects, disturbed growth, multiple congenital organ malformations, as well as hematological and immunological anomalies [15]. Although the variants here studied associate with the development of Kabuki syndrome, they have never been evaluated for their mechanism of dysfunction. Thus, the well-documented genotype-to-phenotype correlation for these variants justifies the purpose of these studies, namely extending this knowledge toward better understanding their mechanisms of dysfunction at the molecular level with atomic resolution.

A wealth of computational tools has been developed for categorizing potentially damaging effects [16]. For example, the popular genomics-based predictors, such as SIFT [17] and PolyPhen2 [18], suggest which DNA/protein sequence alterations may be damaging to the function. Recently, the use of 3D methods for structural bioinformatics has elicited significant attention due to its potential for additional predictive values and mechanistic inference. This field, while in its infancy, is represented by predictors such as Missense3D [19] and Rhapsody [20], which use protein structures and coarse-grained Elastic Network Model (ENM)-based predictions for more specific mechanistic alterations. In the current study, we report a more comprehensive analytical approach that uses a wider set of scores derived from molecular mechanic calculations and molecular dynamics (MD) simulations using the published 3D structure of the catalytic domain of KDM6A [21]. To this end, we have developed an analytical framework that shows how these 3D parameters can identify dysfunction in a subset of KDM6A Kabuki-associated variants (within its catalytic domain). These multi-parametric analyses include protein folding/stability, structural perturbation, primary motions at the residue and tertiary structural level, time-dependent binding energy calculations, and p*K*_a_ shift estimations. We demonstrate that these parameters can identify alterations in KDM6A-specific molecular function. Collectively, our data demonstrate that the Kabuki syndrome variants studied here are damaging to this histone demethylase function by various means, thereby extending our understanding of pathobiological mechanisms underlying this disease at the molecular level. Thus, this new knowledge bears both mechanistic and biomedical relevance.

## Results

### Description of KDM6A enzyme and the dynamic behavior of its catalytic domain

**Fig. 1:**
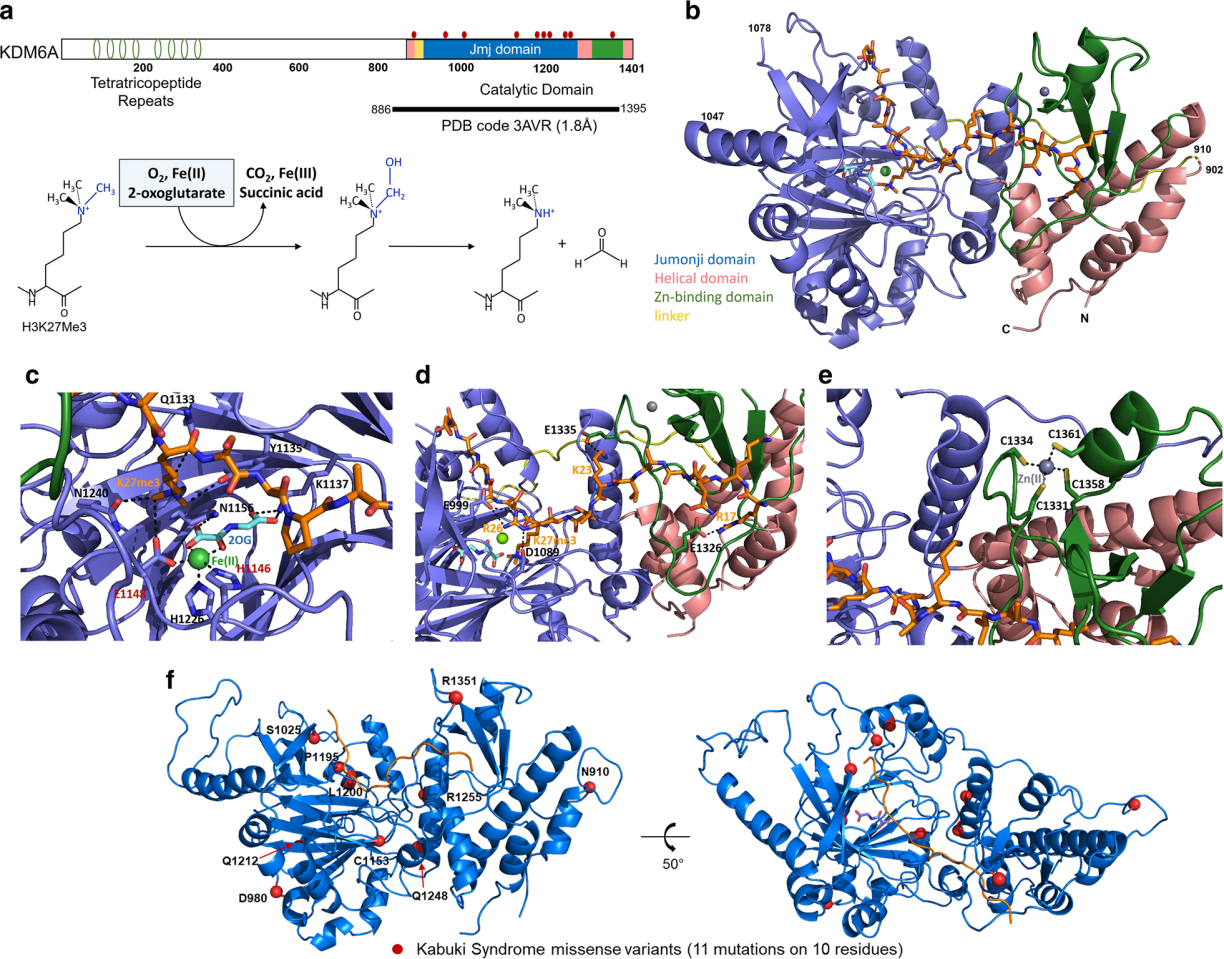
Catalytic mechanism, architecture, key functional sites, and disease-causing missense mutations of KMD6A. **a** Domain structure and the schematic of its catalytic mechanism. Relative positions of *KDM6A* Kabuki variants identified from the patients are highlighted in red on top of the domain diagram. **b** The catalytic domain structure of KDM6A in complex with the H3K27me3 peptide, metal ions and the cofactor 2OG (PDB access code 3AVR). The catalytic domain is composed of the jumonji domain flanked by two additional sub-domains and a long flexible linker. The bound substrate is shown as ball-and-sticks while the catalytic domain is shown as ribbons. The color codes are identical to the ones used in Fig. 1A. (**c**–**e**) Zoomed views of the active site, substrate binding interface, and the zinc ion binding site. two damaging control residues are labeled in red. H3 histone residues are labeled in orange. **f** Mapping of Kabuki syndrome missense variants onto its molecular structure. None of the Kabuki variants are found right at the critical molecular interaction sites. Figures were made using PyMOL (The PyMOL Molecular Graphics System Version 2.3.0., Schrödinger, LLC)

Lysine demethylation involves the iron-dependent oxidation of a methyl group followed by non-enzymatic elimination of formaldehyde (Fig. 1a). KDM6A is made of 1401 amino acids, which separates a linker region that joins two distinctive modular domains; the tetratricopeptide repeat (TPR) domain at the *N*-terminus and the catalytic domain at the *C*-terminus (Fig. 1a). The catalytic domain is comprised of a jumonji domain (residues 933–1047 and 1078–1268, slate blue), a helical domain (residues 886–902, 1269–1314, and 1379–1395, magenta), a proline-rich linker (residues 910–932, yellow), and a Zn-binding domain (residues 1315–1378, green) which plays a critical role in selective substrate recognition (Fig. 1b). The jumonji domain contains a jelly-roll fold flanked by α-helices and represents the most evolutionarily conserved region of the protein (Additional file 1: Fig. S1). The 1.8 Å crystal structure of the catalytic domain in complex with a histone H3K27me3 substrate, a nonreactive cofactor analog *N*-oxalylglycine, a Ni(II) ion at the active site, and the Zn(II) ion coordinated in the Zn-binding domain has been determined and shed light on the molecular basis of its catalysis [21]. Briefly, in its active conformation (H3 peptide-bound), the methylated lysine residue is optimally presented to the active site where the reactive groups are strategically located at specific distances and orientations with respect to the removable methyl groups (Fig. 1c). KDM6A displays maximal activity with the H3K27(me3) which substrate gains a broad ‘W’-shaped conformation during binding (Fig. 1b, d). This bent peptide conformation is stabilized by intra-substrate hydrogen bonds and is required for sequence specificity of KDM6A including sequence-specific ionic interactions such as Glu999-Arg26, Asp1089-Arg26, Glu1326-Arg17, and Glu1335-Lys23 (Fig. 1d). The substrate binding encompasses the buried surface area of 1135 Å^2^, with a polar surface of 294 Å^2^ and 836 Å^2^ nonpolar. In addition, the Zn-binding domain plays a critical role in substrate binding as it goes through a substantial local conformational change when the histone substrate is bound (Additional file 1: Fig. S2). Therefore, any alterations (either direct or indirect) of this critical Zn-coordination site (Fig. 1e) would result in disruption of protein structure and function by negatively affecting the selective substrate recognition and the catalytic activities.

**Fig. 2:**
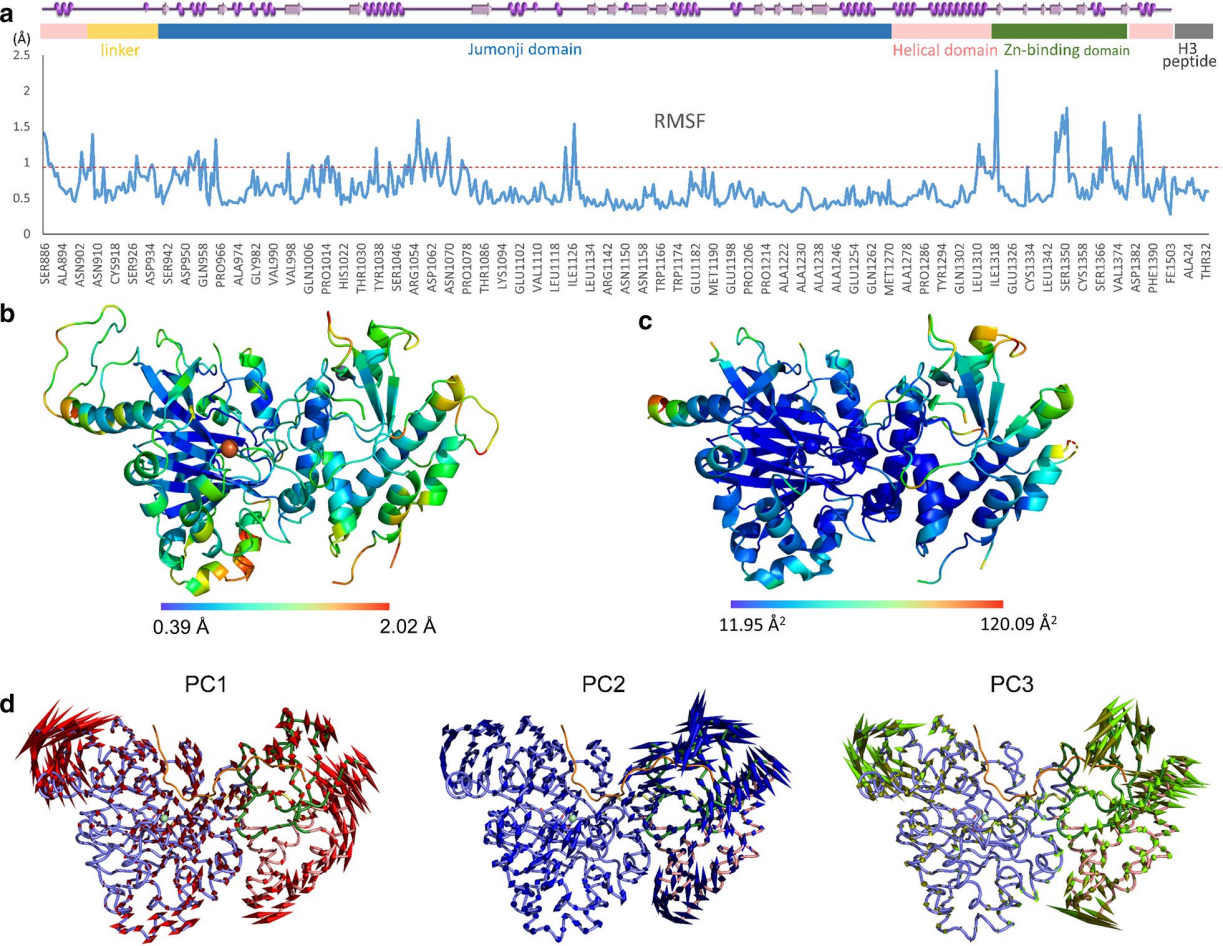
Intrinsic mobility of the KDM6A catalytic domain and the essential dynamic motions. **a** Linear RMSF plot of the KDM6A catalytic domain during MD simulation. The protein regions of greater than 1.0 Å displacements (fluctuations) are indicated by a red dotted line. The secondary structure elements and the domain structures are shown above. **b** Dynamic fluctuations (RMSF) of each residue during MD simulation are also plotted on the molecular structure surface, which is in good agreement of the temperature factor plot of its crystal structure (**c**). They are color-coded according the ranges indicated by the horizontal bar at the bottom of each panel. (**d**) Porcupine plot of trajectories representing the essential dynamic in each principal component during MD simulation. The length of cone represents the motion magnitude and the pointing of the arrow indicates the direction. The catalytic core jumonji domain movements mostly involve PC1 and PC2 while the substrate binding dynamics involve all three main PCs. This plot shows only the enzyme movement

KDM6A, like other proteins, displays an intrinsic dynamic behavior that is uniquely programmed and optimized for its function. Thus, prior to analyzing the mutational impact of Kabuki variants, we probed the dynamic behaviors of the wild type KDM6A catalytic domain through molecular simulations. The initial 10 ns of MD simulation revealed the functionally mobile regions and the essential dynamic motions (Fig. 2 and Supplementary movie M1). The secondary structural features that compose the overall enzyme–substrate complex displayed a coordinated movement while maintaining the compact arrangement of the subdomains. The structural ensemble during the simulation revealed that the core jumonji domain containing the active site remains relatively still (less than 0.7 Å displacement) while the surrounding regions display more active motions. This dynamic behavior complements and extends previous observations that the catalytic mechanism of KDM6A does not require additional mobile regulatory elements near the active site [7]. The substrate recognition dynamics serve as a rate-limiting step since once the substrate is presented to the active site, the active site catalysis of KDM6A readily takes place. The highly mobile regions (> 1.0 Å displacement) include regions near the surface of the jumonji domain, the substrate recognition, the zinc binding domain, and the helical domain with a potential allosteric regulatory function (Fig. 2a–c). The atomic displacements (fluctuations) of individual residues during MD simulations are in good agreement with the temperature factors of the crystal structure (Fig. 2b, c). This suggests that the simulation captures the most common low energy conformations that represent the native molecular ensemble that links structure-to-function in the analyses of KDM6A. Furthermore, the principal component analysis (PCA) of the structural ensemble reveals high-amplitude concerted motions wherein the substrate binding involves the dominant motions of the first three PCs while the movement of the catalytic core jumonji domain involves limited PC1 and PC2 motions (Fig. 2d). Metal ions and cofactors retain their bound state and ideal geometry with the ligating residues throughout the MD simulation. Together, this data describes for the first time a structure-to-dynamic coupling that characterizes the time-dependent molecular behavior of this Kabuki-associated histone demethylase.

### Selection of well-documented, yet rare, Kabuki-associated KDM6A variants for molecular mechanics and MD simulation studies

To test the utility of our biophysically parametrized structural bioinformatic methods [1–4], we selected KDM6A variants within the catalytic domain found in patients with a Kabuki syndrome diagnosis. These have previously predicted to be damaging in most cases (with a few exceptions) by available impact predictors but for which more studies remain necessary to determine if their classification is valid and to identify the mechanisms underlying their dysfunction (Table 1). Nine missense variants were identified through Human Gene Mutation Database (HGMD) [22] and ClinVar [23] (D980V, S1025G, C1153R, C1153Y, P1195L, L1200F, Q1212R, R1255W, and R1351Q). In addition, two other missense variants, N910S and Q1248R, reported in the literature [13, 24], but not deposited in the databases were added to our study. Figure 1f displays the locations of these well-documented eleven Kabuki syndrome missense variants on the molecular structure. We find that no Kabuki variants directly affect molecular interactions with the key functional/structural elements to its enzymatic activity, such as with the cofactors, substrate, and zinc ligation (Fig. 1c–e). However, these interactions could also be allosterically affected by distant mutations in the 3D structure. We used a non-damaging variant (H1060L) as the main control, because it is annotated as ‘benign’ in ClinVar, annotated as SNP in the Single Nucleotide Polymorphism Database (dbSNP), and displays a higher allele frequency (3.07 × 10^–4^) in the general population (gnomAD). As additional benign controls, we used two healthy individual variants from the gnomAD database (S912N and T1323A) which have a higher allele frequency (> 2.0 × 10^–5^). For damaging control variants, we used two non-naturally-occurring variants (H1146A and E1148A) whose deleterious effects and loss-of-function activities are well-documented [25, 26]. We probed the disruptive effect of each mutation by calculating a series of 3D structure-based scores for molecular fitness. These include static structure-based analyses (protein folding energy, protein stability, local conformation, p*K*_a_ values of the ionizable residues), and molecular dynamic-based analyses (root mean square deviation (RMSD), root mean square fluctuation (RMSF), and time-dependent interaction energy calculations). We also compared the 3D methodology with benchmark variant calling methods, commonly used in the practice, such as PolyPhen2 [18] and Rhapsody [27], chosen here because of their improved algorithms and machine-learning capabilities. The results of the analyses by these methods are shown in Table 2. Notably, we find that the N910S and R1351Q Kabuki variants are predicted to be non-damaging by the sequence-based programs, yet potentially damaging by the static structure- and dynamic-based analyses as described below. Thus, our results demonstrate that, although sequence-based tools are useful and convenient, they are not highly dependable as they lack functional mechanism-based interpretations.

**Table 1:** Missense Kabuki syndrome and control variants in KDM6A used on our experiments

Variant	Mutation	Protein Change	Clinical Data	Segregation	Classification	(PMID) and Databases
H1146A		p.(His1146Ala)	NA	NA	Damaging	17,761,849, 18,003,914
E1148A		p.(Glu1148Ala)	NA	NA	Damaging	17,761,849
H1060L	c.3197A > T	p.(His1060Leu)	NA	NA	Benign	24,728,327, ClinVar, dbSNP
S912N	c.2735G > A	p.(Ser912Asn)	GP	NA	Benign	gnomAD
T1323A	c.3967A > G	p.(Thr1323Ala)	GP	NA	Benign	gnomAD
N910S	c.2729A > G	p.(Asn910Ser)	KS ^a^	Inherited	VUS	27,302,555
D980V	c.2939A > T	p.(Asp980Val)	KS	De novo	Pathogenic	24,633,898, 30,107,592, HGMD
S1025G	c.3073A > G	p.(Ser1025Gly)	KS	Inherited	Likely Pathogenic	27,302,555, Clinvar
C1153R	c.3457 T > C	p.(Cys1153Arg)	KS		VUS	ClinVar
C1153Y	c.3458G > A	p.(Cys1153Tyr)	KS	De novo	Likely Pathogenic	ClinVar
P1195L	c.3584C > T	p.(Pro1195Leu)	KS	De novo	Likely Pathogenic	ClinVar
L1200F	c.3598C > T	p.(Leu1200Phe)	KS	Inherited	Likely Pathogenic	ClinVar
Q1212R	c.3536A > G	p.(Gln1212Arg)	KS	Germline	Likely Pathogenic	ClinVar
Q1248R	c.3743A > G	p.(Gln1248Arg)	KS	De novo	Pathogenic	30,107,592
R1255W	c.3763A > G	p.(Arg1255Trp)	KS ^b^	De novo	Pathogenic	27,302,555, ClinVar
R1351Q	c.4052G > A	p.(Arg1351Gln)	KS		VUS	ClinVar

GP, General Population, KS, Kabuki Syndrome

^a^classic KS facial features, intellectual disability; no cardiac/renal dysfunction

^b^mild motor delay, no-to-little cognative disability

**Table 2:**
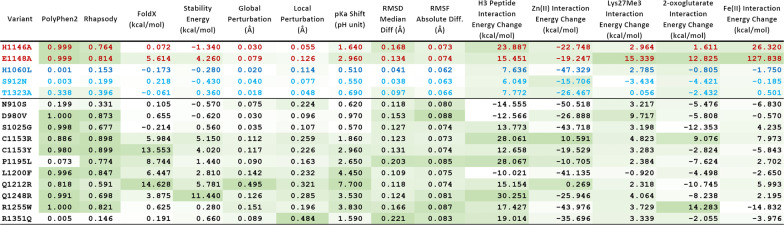
Numerical scores for the Kabuki variants from each sequence, structural and dynamics analysis.

Intensity of the color shadow in each box indicates the level of damaging effect

### Kabuki-associated KDM6A variants encode proteins that show local structure and stereochemical perturbation and chemical environment alterations

Protein folding and stability are foundations of protein function, and their respective energy calculations have been used to assess the potential impact of mutations [28, 29]. For our study, the stability of the mutated protein was assessed by the variant-induced changes in folding energy ($\Delta \Delta {\text{G}}$_fold_) using FoldX [30] and free energy changes using the Discovery Studio suite (Dassault Systèmes BIOVIA). A high degree of agreement between these two independent estimations is observed (Table 2, columns 4–5). The benign controls (H1060L, S912N, and T1323A) are not affected, and one of the damaging controls (H1146A) is only minimally affected, yet its weakening of Fe(II) coordination and the overall structural drift were detected during MD simulation (Table 2). Overall, our data suggest that all Kabuki variants disrupt the wild type-like structure of the KDM6A protein and likely impair the protein function. To function properly, a protein must maintain its overall structural, local structural–functional, and dynamic features. Any amino substitutions that alter local stereochemical environments such as non-bonding interaction patterns, polarity, and steric configurations can be damaging to protein function. To address the global (entire domain) and local (within a 10 Å radius of the mutated residue) perturbation resulting from a missense variant, we used Missense3D [19] for initial impact analysis and subsequently measured atomic displacements between the wild-type and variants from their energy minimized structures using PyMOL (Molecular Graphics System, Schrödinger, LLC) and Coot [31]. The results of these analyses are shown in Table 2 (columns 6–7). Most Kabuki variants are predicted to cause local and/or global perturbations, much greater than those of the benign controls. However, note that, as exemplified by the N910S and R1351Q, some variants are predicted to be non-damaging by sequence-based programs but show significant local structural perturbations by our analyses. Thus, although global perturbations can be informative, local perturbations seem to be more reliable as damaging effect predictors.

**Fig. 3:**
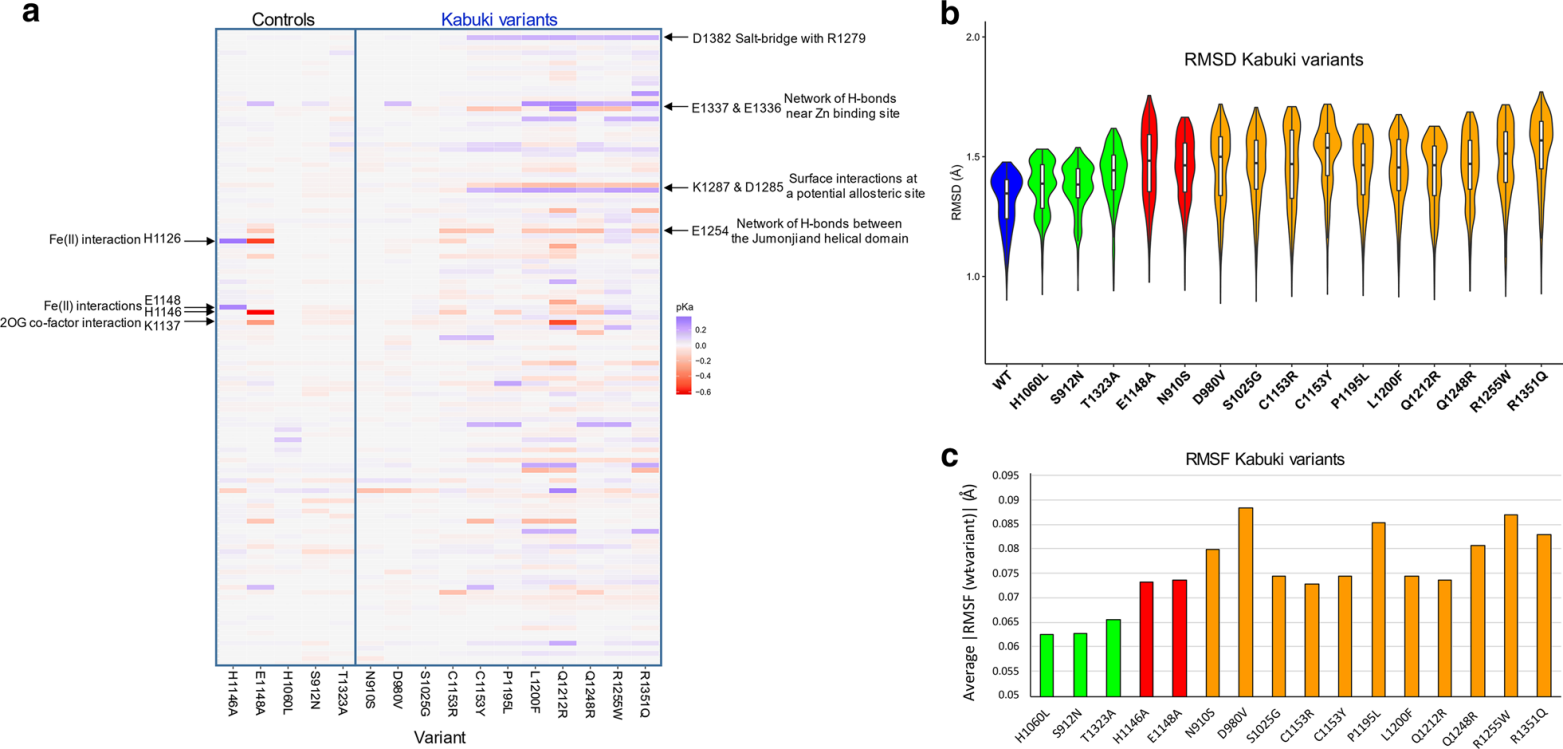
Representative impact analyses. **a** A heatmap representation of the global p*K*_a_ shifts due to each mutation. p*K*_a_ shifts of the residues that are directly affected by the substitution (e.g., H1060 of the H1060L mutant or H1146A, E1148A, R1255W, and R1351Q original residues and newly introduced charged residues such as C1135R, Q1212R, and Q1248R) are not shown and only indirect effects are represented. Shift amounts in pH unit are color-coded (indicator bar on right). The vertical axis shows the list of titratable residues in a descending order from the top. Noticeably affected residues are indicated on both sides; key functional residues on left and potential allosteric residues on right. **b**, **c** MD-based evaluation of global dynamic alterations, such as **b** the comparison of RMSD distribution and median values and **c** the absolute average RMSF differences. Wild type (blue), benign (green), damaging controls (red) and Kabuki variants (orange) are shown. Various comparative values have been tested and ‘Median Difference’ for RMSD and Avg. (|WT-variant|)’ per residue were chosen as the best metrics and plotted for variant impact assessment. All Kabuki variants displayed elevated global motions that deviate from the wild type

Histone lysine demethylases such as KDM6A are oxidative enzymes whose catalytic activities are very sensitive to local pH and oxygen concentration [32, 33]. In particular, an Fe(II) oxidation sate in the 2OG-dependent dioxygenase superfamily members is critical for substrate methyl oxidation process. Any mutations that disrupt the p*K*_a_ of protein residues, including the Fe(II) chelating residues, are expected to affect the overall protein function. To investigate how much titratable residues undergo protonation change upon each mutation, we calculated their p*K*_a_ values using DelPhiPKa [34]. Then, the overall p*K*_a_ shifts were calculated by summing the p*K*_a_ differences of each residue between the controls and Kabuki-associated variants in both directions. We calculated p*K*_a_ shift amounts for the entire catalytic domains to survey the global effects. As shown in Fig. 3a and Table 2 (column 8), the damaging controls (first two lanes of the heatmap and the corresponding values in Table 2) and the majority of Kabuki variants (except the first three, N910S, D980V, and S1025G) caused greater shifts while the benign controls (third to fifth lanes) exhibited minimal shifts in their overall p*K*_a_ values. Our data also indicate that p*K*_a_ shifts are not random, but rather strongly coordinated throughout the protein and certain ‘weak-spots’ and more sensitive residues primarily affected by the disease variants (Fig. 3A). These ‘weak-spot’ residues include key functional residues such as the metal Fe(II) ion and cofactor interaction residues (indicated on left in Fig. 3A) as well as several residues within the helical domain that could affect potential allosteric regulations (indicated on right). The benign controls did not cause any notable changes in the p*K*a values of these ‘weak-spot’ residues. This finding is important, since it indicates that potentially damaging variants can be recognized by this simple parameter. This type of p*K*_a_-based analysis can be applied to other relevant proteins such as catalytic enzymes and pH-sensing proteins.

### Kabuki-Associated KDM6A variants also display altered dynamic behaviors and disrupted functional molecular interactions

We next focused on how variants may affect molecular dynamic-based features of the protein. Consequently, we performed MD simulations to investigate their overall motion and time-dependent molecular interactions as indicators of active site dynamics and substrate binding dynamics. For overall impact scoring, MD trajectory files were analyzed for differences in RMSD and RMSF values. Structural drift (global changes in structure) was measured by monitoring RMSD values from the initial structures as a function of time. We find that the overall structures across variants and replicates were not significantly altered, and the compact conformations were largely preserved in all variants. The majority of variants displayed a median RMSD values around 1.5 Å for all atoms (Additional file 1: Fig. S3). The median difference between each variant and the benign controls were used to assess variant impact (Table 2, column 9). Our results reveal that Kabuki variants show considerable changes in their global dynamics to varying degrees with respect to the wild type, ranging from 8.1 to 16.4% for variants and 2.7 to 6.7% for benign controls; some (such as C1153Y and R1351Q) show even bigger changes than the damaging controls (Fig. 3b). Thus, we believe all Kabuki variants are damaging to protein function as molecular dynamics disruptors.

Additional information was derived at the individual residues level by RMSF calculations, which approximates molecular flexibility. We tested various comparative measurements for developing a RMSF score, such as Spearman correlation coefficient, Pearson correlation coefficient, average difference, average absolute difference, and absolute residual sum, among which average absolute difference was chosen as the best metric for variant impact assessment (Additional file 1: Fig. S4) and shown in Table 2 (column 10). Data distribution is also graphically represented in Fig. 3c. All Kabuki variants also show elevated RMSF values (12 to 19.6% increase compared to 5.7 to 6.0% increase for the benign controls); many (such as N910S, D980V, P1195L, Q1248R, R1255W, and Q1351Q) show even bigger elevations than the damaging controls (Fig. 3c). We also performed principal component analysis (PCA) to quantify differences in essential molecular motions associated with each variant. This was done by displaying the MD structural clusters of each variant onto the bidimensional free-energy landscape (FEL) described by the principal components and measuring the distributional shifts in each direction with respect to the wild type (Additional file 1: Fig. S5). However, the results show that these measurements are not very congruent with other scores, indicating that this analysis might represent a novel approach that needs to be interpreted independently or more effective PC-based measurement schemes need to be devised.

The activity and selectivity of KDM6A is related to the binding affinities of the enzyme to the reactive groups in the active site and to its substrates. Thus, we measured the magnitude of changes in time-dependent interaction energies for key molecular interactions at these sites for each variant using the protocol implemented in the Discovery Studio. For substrate interactions, we calculated the energies for each the peptide and the Lys27me3 residue alone. As shown in Table 2, the metal ion binding disrupting controls (H1146A and E1148A) show destabilization of Fe(II) interactions as predicted, while other variants have minor or even stabilizing effects. Note that the disturbance by the metal-binding controls have even further rippling effects on neighboring key reactive group interactions with cofactors indicate that multiple interactions at the active site could be coupled. More importantly, Table 2 shows that many Kabuki variants (such as D980V, S1025G, C1153R, C1153Y, P1195L, Q1212R, Q1248R, R1255W and R1351Q) have negative effects on specific interactions. The C1153R variant is predicted to affect dynamic interactions with all three key components, namely cofactors, substrate, and Zn(II) ion. These data indicate that although mutations occur on non-interacting residues, the disease variants can have remote effects on the key functional and structural interactions, hinting potential allosteric regulations.

### Underlying mutation-specific mechanisms of dysfunction derived from molecular mechanics calculations and dynamics simulations

To provide variant-specific comprehensive interpretations, potential damaging effects of each Kabuki-associated variant and its disease-associations are elaborated herein. The overall impact predictions based on our analyses are summarized in Table 3.

**Fig. 4:**
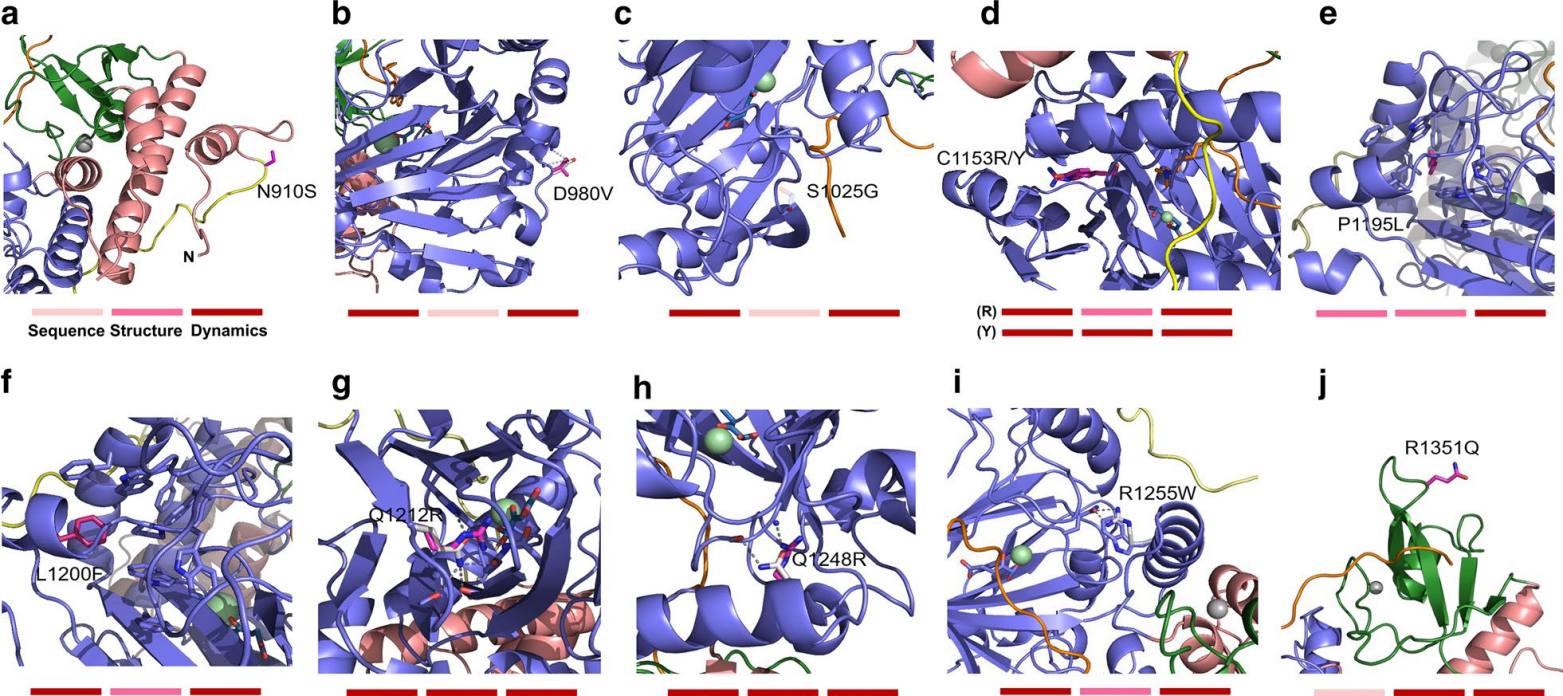
Close-up views of Kabuki variants under study and their category-wise assessment. Further descriptions on the alterations of local environment and chemical interactions by these mutations are provided in the main text. At the bottom of each panel, colored boxes represent overall assessment of each category for each Kabuki syndrome variant; sequence-, structure-, and MD-based, respectively from left to right. The intensity of redness indicates the level of damaging impacts at each layer light to dark. Although N910S and R1351Q are predicted to be non-damaging by sequence-based analysis, they are predicted to be damaging by static structure- and dynamics-based mechanistic analyses. There is a set of indicators for C1153 representing C1153R (top) and C1153Y Kabuki variants

1. KDM6A N910S: The N910 residue is located at the beginning of the flexible linker connecting the helical to the jumonji domain (Fig. 4a). This residue is fully exposed to solvent and a N to S substitution is predicted to have very little damaging effect to the protein by PolyPhen2 and Rhapsody (Table 2). However, our data indicate that this variant can cause local perturbation (0.2238 Å on average within 10 Å radius) and a drift in molecular dynamics. This variant is unlikely to impact protein folding/stability nor molecular interactions used for catalysis (Table 2). This variant is inherited and the patient exhibits classical Kabuki syndrome facial features and intellectual disability.
2. KDM6A D980V: The D980 residue is located at the flaking region of jelly-roll fold of the jumonji domain (Fig. 4b) and leads to the formation of hydrogen bonds with the backbone amide nitrogen atoms of L981 and G982. Our data predicts that a D to V substitution causes a loss of these hydrogen bonds thereby disturbing the local protein-solvent interface as indicated by considerable differences in RMSD and RMSF values, thus acting as a molecular dynamics disruptor (Table 2 and Fig. 3b, c). The variant shows little changes in folding energy and stability (0.655 and − 0.62 kcal/mol, respectively), but is expected to remotely affect the specific interaction between KDM6A and the Lys27Me group of the substrate (Table 2).
3. KDM6A S1025G: This amino acid substitution is located near the jelly-roll fold of the jumonji domain and the H3 substrate binding interface (Fig. 4c). In the variant, glycine forms a single hydrogen bond with the neighboring Q1003 but, besides this small change, other structure-based parameters predict low damaging effects. However, MD simulation analysis indicates that protein dynamics can be disrupted with a weakened substrate recognition (Table 2).
4. KDM6A C1153R: This residue is located within the core jelly-roll fold of the jumonji domain near the active site (Fig. 4d). The surrounding local structure is tightly packed and the C to R substitution introduces a buried charged residue that significantly disrupts local conformation, including at the active site (Table 2 for protein stability, local perturbation, and molecular dynamics). As a result, key functional interactions such as 2OG and substrate interactions are disrupted, and the zinc-ligation is also affected (Table 2).
5. KDM6A C1153Y: This variant can perturb the local structure leading to a large shift in folding energy/stability and p*K*_a_ as well as affect global dynamics parameters with the disruption of protein-substrate interactions (Table 2). This variant has also been found as a somatic mutation in colorectal carcinoma (COSMIC ID: COSM6692688) [35, 36].
6. KDM6A P1195L: This variant, which has also been found as a somatic mutation in esophageal adenocarcinoma (COSMIC ID: COSM1255502) [37], is located within the hydrophobic core of the jumonji domain at the beginning of an α-helical segment (Fig. 4e). Although PolyPhen2 predicted a low damaging effect (Table 2), a P to L substitution at this site is predicted to alter the backbone geometry (disallowed phi/psi) and cause significant steric hindrance. In addition, our data support predicts the structure-to-functional disruption consistent with various biochemical parameters (folding/stability, local perturbation, p*K*_a_ shift, and substrate recognition) as well as alterations in its molecular dynamics (Table 2 and Fig. 3b, c).
7. KDM6A L1200F: The affected residue is located within the hydrophobic core of the jumonji domain which provides a proper microenvironment but the increased size of this substitution (Fig. 4f) likely causes local steric hindrance and thereby disturbing local structure, folding/stability energies, and p*K*_a_ shift (Table 2).
8. KDM6A Q1212R: This variant locates within the core jelly-roll fold of the jumonji domain near the active site and forms a hydrogen bond network with neighboring residues, N1156 and W1166 (Fig. 4g). Although a Q to R substitution retains some of the wild type interactions, the positive charge and increased size of arginine are predicted to perturb local structure and protein folding/stability energies (Table 2). Notably, this variant shows the largest shifts in folding energy, global perturbation, and p*K*_a_ values (Table 2 and Fig. 3a). Additional evidence suggests that this amino acid substitution can also impact substrate recognition (Table 2).
9. KDM6A Q1248R: This amino acid change occurs at a conserved region near the jumonji domain and leads to a gain in a pair of hydrogen bonds with the backbone carbonyl oxygen and amino nitrogen (Fig. 4H). Like Q1212R, this substitution could perturb local structure and greatly reduce protein stability (Table 2). This variant is also expected to cause a considerable structural drift and weakened substrate interactions (Table 2 and Fig. 3b).
10. KDM6A R1255W: This variant has also been found as a somatic mutation in a number of different cancer types (COSMIC ID: COSM212434) [38, 39]. This amino acid change falls within a critical interface between the jelly-roll fold and the adjacent helix (highly conserved and less mobile element) within the jumonji domain (Fig. 4i). A drastic R to W substitution is predicted to cause multiple consequences including local/global perturbation, p*K*_a_ shift, and structural drift by altering the chemical/spatial environment as well as disruption of a hydrogen bond with Q1147 (Table 2). Furthermore, this variant may perturb the 2OG cofactor interaction (Table 2).
11. KDM6A R1351Q: This variant has also been found as a somatic mutation in neurofibromatosis and caecum carcinoma (COSMIC ID: COSM6952414) [39, 40]. This residue is located within a zinc-binding domain (highly mobile region) and the main chain and side chain are fully exposed to solvent (Fig. 4j). Although this variant is predicted to have minimal impact by both PolyPhen2 and Rhapsody (Table 2). However, this result is confounded by the fact that the R1351 residue is not strictly conserved and the same glutamine residue is found at this position in KDM6C (Additional file 1: Fig. S6), rendering this algorithm of low accuracy. Notably, however, we find that this variant displays the largest amount of local perturbation and structural drift from the starting model during MD simulation (Table 2). Thus, this substitution likely has a damaging effect on these proteins and may be in part responsible for low enzymatic activity of KDM6C [41].

**Table 3:** Overall impact prediction of Kabuki variants derived from the assessment at each layer. Impact assignment of the variants at each layer are tentatively given by referring to the control values. These data illustrate the multiple pathways these variants may be affecting KDM6A function

Variant	Sequence	Structure	pK Shift	Dynamics	Substrate and Zn interaction	Active site interactions	Overall impact prediction
H1146A	Damaging	Benign	Damaging	Damaging	Damaging	Damaging	Damaging control
E1148A	Damaging	Damaging	Damaging	Damaging	Damaging	Damaging	Damaging control
H1060L	Benign	Benign	Benign	Benign	Benign	Benign	Benign control
S912N	Benign	Benign	Benign	Benign	Benign	Benign	Benign control
T1323A	Benign	Benign	Benign	Benign	Benign	Benign	Benign control
N910S	Benign	Damaging	Benign	Damaging	Benign	VUS	Damaging
D980V	Damaging	VUS	VUS	Damaging	Benign	Damaging	Damaging
S1025G	Damaging	VUS	Benign	Damaging	Damaging	VUS	Damaging
C1153R	Damaging	Damaging	Damaging	Damaging	Damaging	Damaging	Damaging
C1153Y	Damaging	Damaging	Damaging	Damaging	Damaging	VUS	Damaging
P1195L	VUS	Damaging	Damaging	Damaging	Damaging	VUS	Damaging
L1200F	Damaging	Damaging	Damaging	Damaging	Benign	Benign	Damaging
Q1212R	Damaging	Damaging	Damaging	Damaging	Damaging	VUS	Damaging
Q1248R	Damaging	Damaging	Damaging	Damaging	Damaging	VUS	Damaging
R1255W	Damaging	Damaging	Damaging	Damaging	Damaging	Damaging	Damaging
R1351Q	Benign	Damaging	Damaging	Damaging	Damaging	VUS	Damaging

## Discussion

In this study, we applied comprehensive sequence-, structure-, dynamic-based approaches to study the potential mechanisms by which disease-associated missense variants may affect KDM6A enzymatic activities. Eleven Kabuki syndrome missense variants within the catalytic domain, along with five selected controls, were chosen and characterized for structure- and dynamics-based impact prediction to gain molecular insight into how these genetic variations may affect KDM6A protein function. Among the set of variants, we have shown many types of mechanistic disruptions in folding, global and local structural perturbation, p*K*_a_ shifts and interactions with the histone H3, cofactors and metal ions that would contribute to a damaging effect on the protein.

Although all Kabuki syndrome missense variants are found in non-critical functional regions, our data indicate that they all significantly disrupt the global and local conformation, chemical environment, and/or molecular dynamics, and some remotely affect the critical molecular interactions. For the benign control variants (H1060L, S912N, and T1323A), all the measures consistently support that these substitutions can be tolerated while evident damaging effects for H1146A and E1148A were clearly demonstrated. Three variants identified in Kabuki Syndrome patients, N910S, P1195L and R1351Q, were not predicted to be damaging by at least one sequence-based score. However, by considering the structural and dynamics-based scores, we have identified many mechanisms by which these Kabuki syndrome variants may damage the function of KDM6A. For example, structural score FoldX shows that P1195L is destabilized relative to the wild-type protein, while P1195L and R1351Q have considerable p*K*_a_ shifts, and all three variants have elevated global and local perturbations. When using dynamics-based scores, all three variants exhibited potentially damaging changes through increased atomic displacement and destabilization of the protein. Although all three show only subtle disruption of active site key interactions, P1195L and R1351Q show significantly destabilized substrate interactions. These data indicate that structural and dynamics-based analyses are essential for more accurate impact assessment. All Kabuki syndrome variants are damaging to protein function as they all prove to be molecular dynamics disruptors (MD variants) and the majority also affect the structural aspects of the protein (structural variants) that are directly related to protein’s integrity and critical function. Although all Kabuki variants are located at lesser mobile regions (< 0.7 Å displacement), except R1351Q (1.21 Å displacement), their mutations are expected to greatly affect protein’s functional dynamics and overall activities.

Kabuki syndrome is a rare disease which occurs about once in every 32,000 births and KDM6A mutations account for only ~ 5% of all cases. Because case reports with KDM6A missense mutations are very limited, we cannot make any conclusive and scientifically sound statements on the prevalence of the mutations. Moreover, most mutations are truncation/deletion/nonsense/frame shift or regulatory mutations such as splice site and non-coding DNAs. D980V was the only one reported more than once. On the other hand, there are plenty of KDM6A cancer somatic missense variants that are found in the database and four of the Kabuki-associated variants (C1153Y, L1200F, R1255W, and R1351Q) have been also identified from cancer patients. Although C1153Y, L1200F, and R1351Q have been reported only once, the most ‘damaging’ variant R1255W (Tables 2, 3) has been reported in an exceptionally high number of 12 times from various cancer types such as stomach, prostate, and uterus cancers. This might suggest that more damaging variants can be more frequently found in human diseases. However, the prevalence of Kabuki germline variants cannot be currently addressed with the limited information.

Various metrics used to analyze MD trajectories can elucidate different properties of protein macromolecules, such as protein stability, energy distribution, and functional flexibilities. For the current study, we used RMSD, RMSF, and time-dependent molecular interactions as reliable MD-based metrics that are effective in quantifying alterations of dynamic properties associated with each variant. Additional structure-related metrics such as protein surface features [20], four-body contact potential [42] and local energetic frustration [43] can be further used for more in-depth analysis in a protein-specific manner. We also found that, among the structure-based metrics, p*K*_a_ shift analysis was particularly useful for KDM6A and should be applicable to other related proteins.

Some KDM6A variants may additionally affect other aspects of protein life cycle and protein function such as gene expression, RNA dynamics, protein translocation, protein–protein interactions, and post-translational modification. In particular, surface residues such as N901 and R1351 could affect protein–protein or protein-DNA interactions as this protein is present in a complex that contains additional histone modifying enzymes such as CBP/p300, chromatin remodeling SWI/SNF proteins, and KMT2C/D and may contact DNAs that are associated with nucleosome [36, 44]. KDM6A also interacts with mixed-lineage leukemia complex 3 (MLL3) and MLL4 [45]. In fact, impaired H3K4 methyltransferase activity of KMT2D variants, another Kabuki syndrome associated gene product, is in part due to disruption of WRAD complex formation, which further associates and stimulates the COMPASS catalytic activity [24]. Thus, we cannot rule out the possibility that KDM6A Kabuki syndrome variants have multifarious damaging effects beyond what we present in the current study.

## Conclusions

Overall, our study provided a molecular insight into how KDM6A disease variants might disrupt the function. These data reinforced the fact that Kabuki variants are loss-of-function mutations, and protein structure and dynamics are essential elements for protein’s optimal function and normal physiology. Our data clearly indicate that the comprehensive assessment, including molecular dynamics, is superior to the present annotation tools used in human genomics databases. Our comprehensive approaches add another layer to the overall damaging impact interpretation that is more directly related to protein’s molecular function. We believe that MD simulations can become an integral part of meta-prediction approaches which can improve the accuracy and sensitivity of damaging effect prediction for interpretation of genomic data derived from next generation sequencing. The time-dependent, three-dimensional dynamic behaviors should add value to sequence- and static structure-based analyses and allows more detailed inference and mechanistic predictions to be made. The widespread adoption of these methods can provide better diagnosis, risk assessment, and clinical guidelines for the observed variants within the context of individualized medicine.

## Methods

### Preparation of the initial structure

High resolution (1.8 Å) crystal structure of a histone H3K27me3 peptide [17–33]-bound form of human KDM6A catalytic domains (PDB access code 3AVR) was used in our study. This structure contains a Ni(II) ion (instead of the enzymatic Fe(II) ion) and the cofactor analog *N*-oxalylglycine at the active site as well as a structural zinc ion in the zinc binding domain. However, for our studies, the cofactor analog and the Ni(II) ion were replaced with the natural cofactor 2-oxoglutarate (2OG, also known as α-ketoglutarate) and the Fe(II) ion, respectively, to reconstruct the native-like active conformation. In addition, this structure is missing two loop regions (amino acids 902–910 and 1047–1078) due to high mobility, and they were filled in for our analysis using the Modeller program [46]. For missense variant analysis, substitutions were made within the Discovery Studio suite version 19.1 (Dassault Systèmes BIOVIA) by mutating the corresponding residue and selecting the side chain rotamer causing the least steric hindrance with the surrounding residues.

### Protein folding energy and stability calculation

Mutagenesis and structure-based predictions of changes in folding energy ($\Delta \Delta {\text{G}}$_fold_) were computed using FoldX [30]. In addition, shifted amounts of protein stability (free-energy difference between folded and unfolded states) due to mutations were calculated at pH 7.4 within the Discovery Studio using the energy-minimized wild type structure (H3-unbound form) and introducing each substitution for calculation. After the preparation phase, the initial structures of the wild type and the generated mutants were subjected to a two-stage minimization process before subjected for energy calculation. Proteins vary in stability, but generally a ΔΔG in the range of 2 kcal/mol is considered to result in a mutational ‘hot-spot’ of sufficient effect [47].

### Local structure perturbation measurement

From the energy-minimized structures, any residues that reside within 10 Å radius from the mutation site were selected and calculated for the RMSD of the backbone atoms between the wild type and the mutant using PyMOL (Molecular Graphics System, Schrödinger, LLC) and Coot [31]. For global structure perturbation, entire backbone atoms were used for RMSD calculation between the structures.

### p***K***_a_ shift analysis

To investigate the possibility that some titratable residues may undergo protonation change upon single amino acid substitution of KDM6A, we performed the p*K*_a_ calculations at pH 7.0 with DelPhiPKa [34] which is a surface‐free Poisson‐Boltzmann based approach to calculate the p*K*_a_ values of protein ionizable residues. We first calculated the p*K*_a_ values of titratable residues for the wild type and each mutant using energy-minimized structures. The p*K*_a_ shifts then were calculated by subtracting the p*K*_a_ values of the wild type and the mutant residues in both directions and summing up the differences. Loss or gain of titratable residues at the position where Kabuki mutations occur and could dominate the cumulative p*K*_a_ shift amount were not considered in calculations. The R studio code used to generate the p*K*a shift heatmap is provided as a supplementary material (Additional file 1: Text S1).

### Molecular simulations

MD simulations were performed using the CHARMm36 all-atom-force-field [36] implemented in the Discovery Studio with a 2 fs time step. A simplified distance-dependent implicit solvent environment was used with a dielectric constant of 80 and a pH of 7.4, and no further parameterization of a non-standard residue (K27me3), cofactor and metal centers. All MD simulations were carried out using periodic boundary conditions. Models were energy minimized for 5,000 steps using steepest decent followed by 5,000 steps of conjugate gradient to relax the protein structure that was obtained under the stressed crystal environment. Each system of 10 replicates of wild type and each variant was independently heated to 300 K over 200 ps and equilibrated for 500 ps followed by 10 ns production simulation under NPT ensemble by changing the initial seed (100 ns total). Structures during unconstrained dynamics simulation were recorded every 10 ps to give a total of 1000 frames for analyses. For final data analysis, one or two outliers (in some cases none) from each data set of 10 replicates that clearly deviate from the rest in RMSD plots and might represent the minor and rarer form of conformations (altogether 12% of the entire data) were excluded from averaging, and only the last 500 frames that have reached the near minimum total energy state were used. For RMSD/RSMF analysis, all MD trajectories were processed using the tools available within the Discovery Studio and the algorithms implemented in Microsoft Excel program. Different comparative values between the plots were tested and ‘Median Difference’ for RMSD and Avg. (|WT-variant|)’ per residue were chosen as the best metrics for variant impact assessment. The R studio code used to generate the RMSD violin plot is provided as a supplementary material (Additional file 1: Text S1). For PCA, the Bio3D program [48] and the in-house workflow were harnessed. For a porcupine plot representation (Fig. 2D), we derived a matrix **Λ** by diagonalizing *C*_*i*,*j*_ with a transformation matrix **T**, so that **Λ** = **T**^T^**CT**. The columns of **T** are then the eigenvectors **v**_*i*_ of the motion, with the first column being the most significant motion (PC1) and the second (PC2) and the third (PC3), and the diagonal elements of **Λ** are the eigenvalues of the decomposition. A cone drawn from the atomic coordinate of an atom (later combined and averaged for the entire amino acid), with height and direction derived from the components of **v**_1_ that relate to that atom, gives a graphical representation of the motion held in **v**_1_, the first eigenvector, **v**_2,_ and **v**_3._

### Time-dependent interaction energy calculation

Molecular interaction free energies were measured using the protocol implemented in the Discovery Studio. This was done using the MD simulation trajectories. Non-bonded interactions were monitored and dynamic interaction energies (van der Waals and electrostatic energies) were calculated from using the CHARMm36 force field and the implicit distance-dependent dielectric solvent model.

### Overall impact classification of the variant

In Table 3, we tentatively label ‘benign’, ‘VUS’ (variant of uncertain significance), or ‘damaging’ simply by referring to the control values. Anything below the highest value of the benign controls would be considered as ‘benign’ in each category. Anything under the twice value would be considered as ‘uncertain’ and anything higher than that would be ‘damaging’. Also, for each layer (structure, p*K*a shift, dynamics, or time-dependent molecular interactions), there are more than one metrics (except p*K*a shift) and they all need to be considered for collective labeling. Any variants that are predicted to be ‘damaging’ at any layers would be ultimately classified as ‘damaging’. There have been pathogenicity thresholds or mutation significance cutoffs suggested by each predictor or the combination of several predictors; however, they still have a high false positive/negative rate. More quantitative overall scoring schemes or a machine learning model will be considered when we have enough training sets for KDM6A and when we are ready to validate the results.

## Supplementary Information

**Additional file 1**. Supplementary information.

## Data Availability

MD Simulation data will be available upon request.

## Abbreviations

2OG: 2-Oxoglutarate
COSMIC: Catalog of somatic mutations in cancer
dbSNP: Nucleotide Polymorphism Database
ENM: Elastic Network Model
FAD: Flavin adenine dinucleotide
FEL: Free-energy landscape
HGMD: Human Gene Mutation Database
HMT: Histone methyltransferase
IBEM: Inborn error of metabolism
KDM6A: Histone lysine(K)-specific demethylase 6A
MD: Molecular dynamics
PCA: Principal component analysis
PDB: Protein data bank
RMSD: Root mean square deviation
RMSF: Root mean square fluctuation
SNP: Single-nucleotide polymorphism
TPR: Tetratricopeptide repeat
VUS: Variant of uncertain (unknown) significance
