# Correction to: Molecular mechanics and dynamic simulations of well-known Kabuki syndrome-associated KDM6A variants reveal putative mechanisms of dysfunction

**DOI:** 10.1186/s13023-021-01892-4

**Published:** 2021-06-01

**Authors:** Young-In Chi, Timothy J. Stodola, Thiago M. De Assuncao, Elise N. Levrence, Swarnendu Tripathi, Nikita R. Dsouza, Angela J. Mathison, Donald G. Basel, Brian F. Volkman, Brian C. Smith, Gwen Lomberk, Michael T. Zimmermann, Raul Urrutia

**Affiliations:** 1 Genomic Sciences and Precision Medicine Center (GSPMC), Medical College of Wisconsin, Milwaukee, WI USA; 2 Bioinformatics Research and Development Laboratory, and Precision Medicine Simulation Unit, GSPMC, Medical College of Wisconsin, Milwaukee, WI USA; 3 Division of Research, Department of Surgery, Medical College of Wisconsin, Milwaukee, WI USA; 4 Division of Pediatric Genetics, Department of Pediatrics, Medical College of Wisconsin, Milwaukee, WI USA; 5 Department of Biochemistry, Medical College of Wisconsin, Milwaukee, WI USA; 6 Department of Pharmacology and Toxicology, Medical College of Wisconsin, Milwaukee, WI USA; 7 Clinical and Translational Sciences Institute, Medical College of Wisconsin, Milwaukee, WI USA

## Correction to: Orphanet J Rare Dis (2021) 16:66 10.1186/s13023-021-01692-w

After the publication of the original article [1], the authors became aware of the work by Petrizzelli et al., 2020 [2]. The two studies truly represent independent works, for which contents are different. Indeed, although both studies contain molecular dynamics data, each of them use different approaches as well as derived sets of analysis and interpretations which are different in depth of information. Therefore, both studies should be considered complementary to each other.
