# Supplementary material for: Molecular mechanics and dynamic simulations of well-known Kabuki syndrome-associated KDM6A variants reveal putative mechanisms of dysfunction
Source: Orphanet J Rare Dis. 2021 Feb 5;16:66. doi: 10.1186/s13023-021-01692-w (PMC7866879; doi:10.1186/s13023-021-01692-w)
Supplement: Supplementary file 1 — Additional file 1. Supplementary information. [file 13023_2021_1692_MOESM1_ESM.docx]

**Supplementary information**

**Molecular mechanics and dynamic simulations of well-known Kabuki syndrome-associated KDM6A variants reveal putative mechanism of dysfunction**

Young-In Chi, Timothy J. Stodola, Thiago M. De Assuncao, Elise N. Levrence, Swarnendu Tripathi, Nikita R. Dsouza, Angela J. Mathison, Donald G. Basel, Brian F. Volkman, Brian C. Smith, Gwen Lomberk, Michael T. Zimmermann, and Raul Urrutia

**Supplemental movie**

**Movie M1.** KDM6A catalytic domain molecular dynamics simulation (10 ns) animation. The bound-H3 peptide, cofactor, and metal ions are shown as ball and stick models while the protein is shown as ribbons.

**Supplemental figures**

**Figure S1**

**Figure S2**

**Figure S3**

**Figure S4**

**Figure S5**

**Figure S6**

**Supplemental text**

**Text S1. R studio codes used for generating the figures**

**Figure S1**


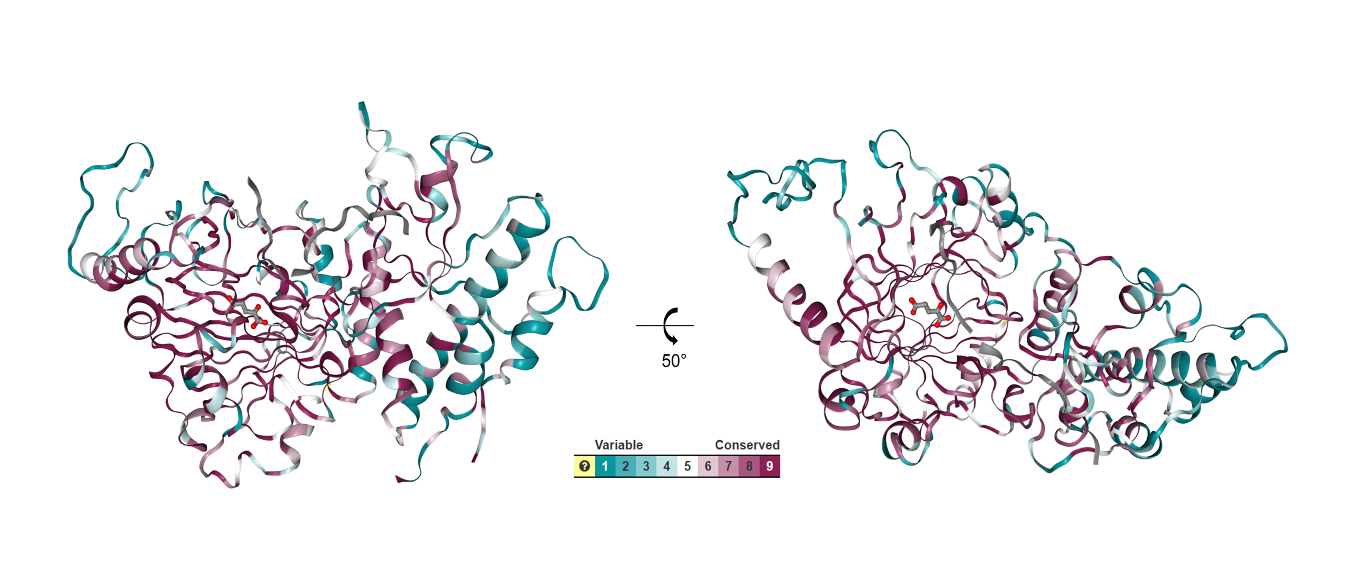


**Figure S1.** Evolutionary conservation of the KDM6A catalytic domain. Identification of integral functional regions by displaying the evolutionary conservation profiles. Conserved regions are shown in purple while variable regions are shown in turquoise. The core jelly-roll fold of the jumonji domain (containing the active site) and the surrounding elements are the most conserved region of the protein. The bound H3 peptide and the 2OG cofactor are shown in grey. This output was generated from the ConSurf server (https://consurf.tau.ac.il).

**Figure S2**


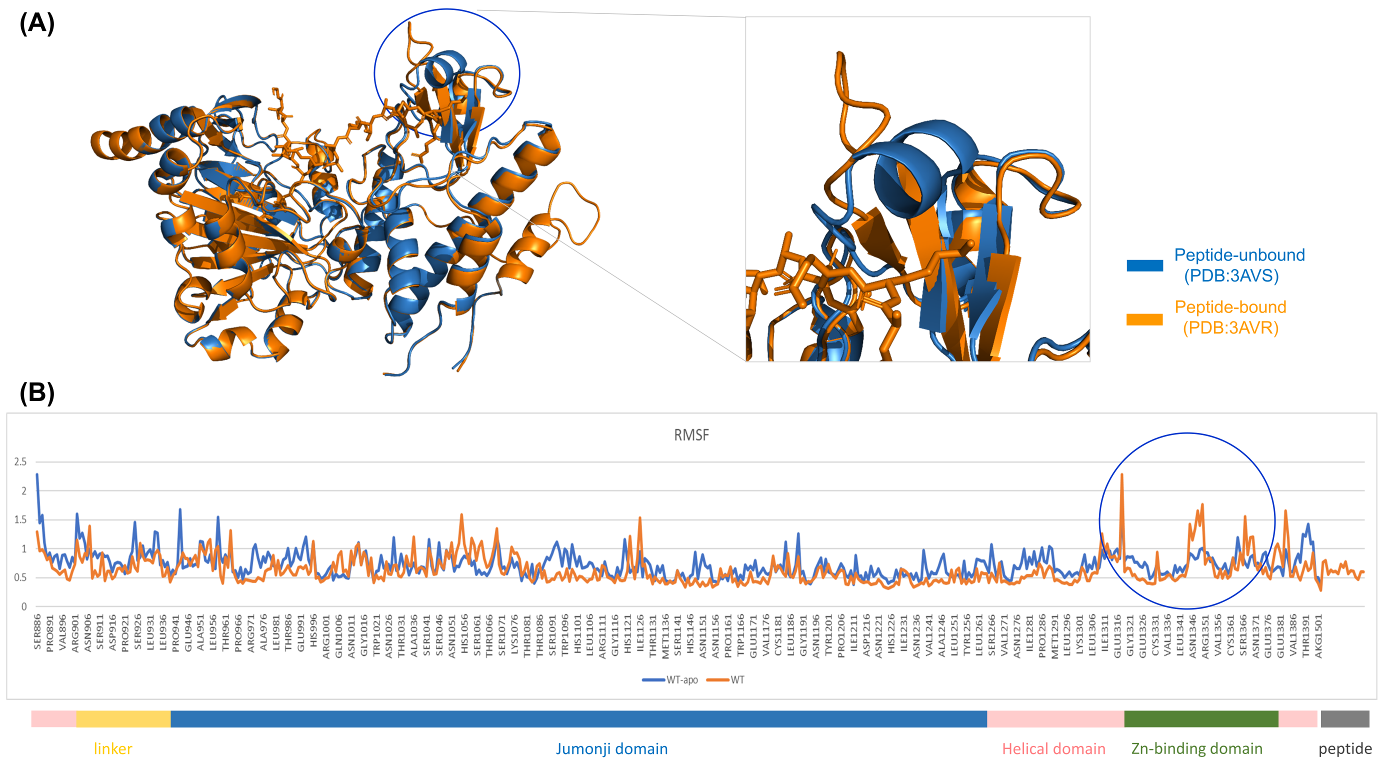


**Figure S2.** Conformational change of the zinc-binding domain to accommodate the H3 peptide. (A) Superposition of the H3 peptide-unbound (blue) and bound (orange) structures (PDB access codes 3AVS and 3AVR). Structural changes are most visible in the zinc-binding domain (blue circle) and the rest of structures are nearly identical. (B) RMSF plots of the trajectories per each residue during the MD production stages of both structures. The most mobile region corresponding to the zinc-binding domain is also circled.

**Figure S3**


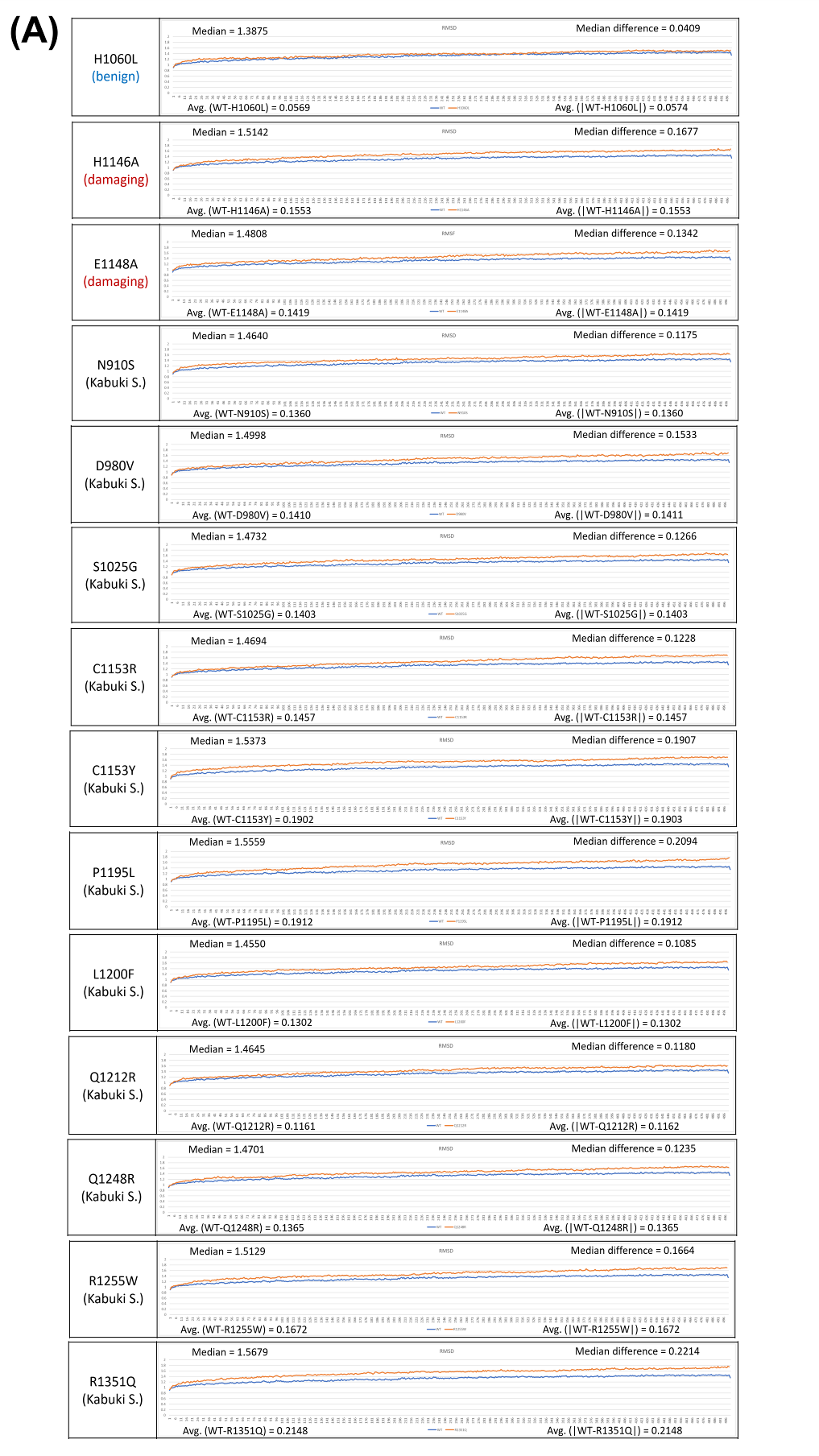


**Figure S3.** Superposition of time-dependent RMSD plots of the wild type (blue) and each variant (orange) for all atoms during the MD production stage. The averaged values of 10 replicates using the last 500 frames are plotted against each cycle. Different comparative values between the plots are indicated around the plots among which ‘Median Difference’ was chosen as the best metric for variant impact assessment.

**Figure S4**


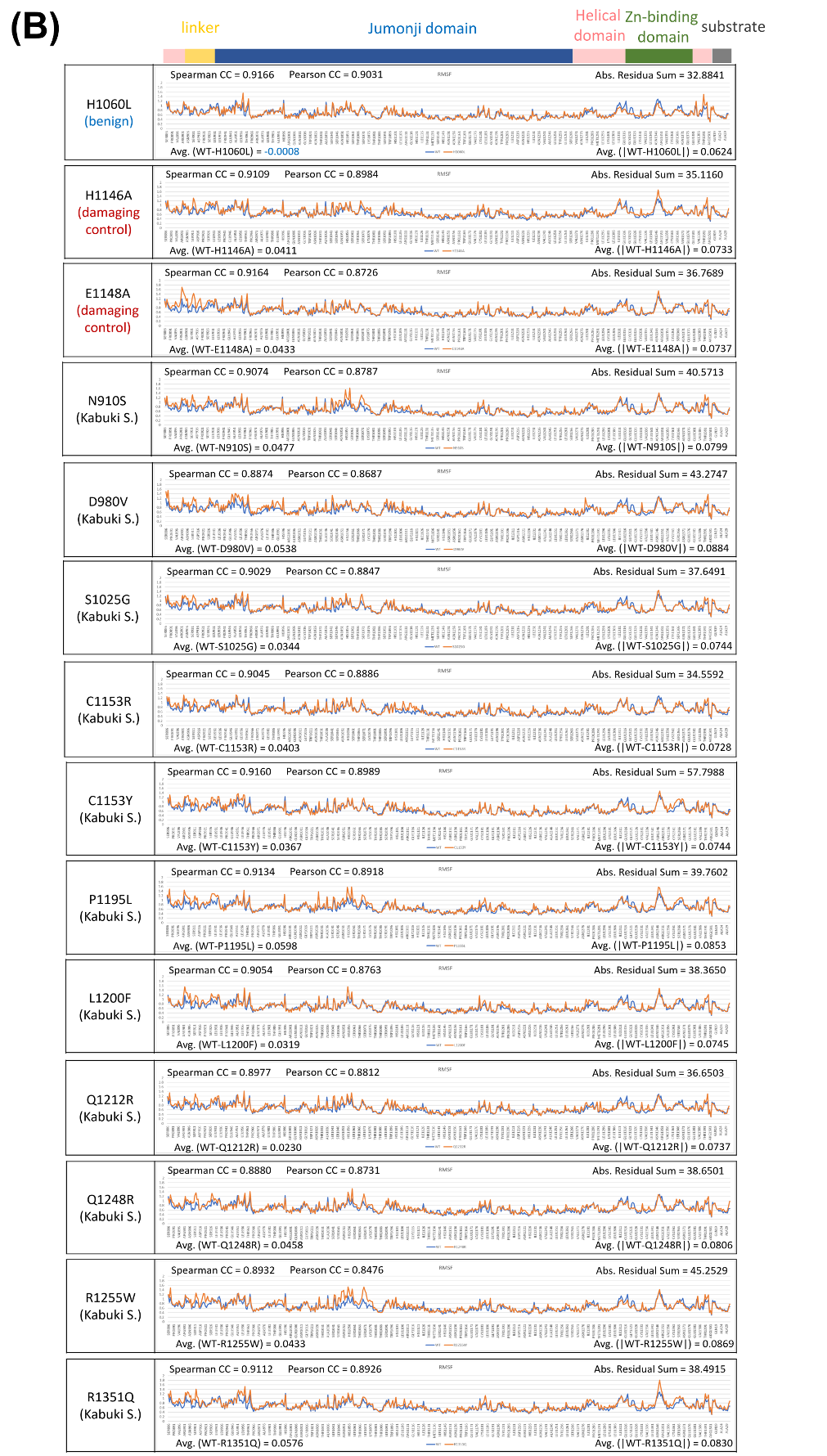


**Figure S4.** Superposition of RMSF plots of the wild type (blue) and each variant (orange) per individual residues during the MD production stage. Also, the averaged values of 10 replicates using the last 500 frames were used. Various comparative measurements between the plots such as Spearman correlation coefficient, Pearson correlation coefficient, average difference, average absolute difference, and absolute residual sum are shown, among which ‘Avg. (|WT-variant|)’ (average difference) per residue was chosen as the best metric for variant impact assessment. The sub-domain structure of the KDM6A catalytic domain is shown on top.

**Figure S5**

**Figure S5.** Principal component (PC)-based impact measurement of Kabuki-associated variants. (A) Structural clustering and their distributional shifts by mutations shown by free-energy landscape (FEL) at 300 K that is projected onto the bidimensional space described by the principal components. The free energy is given in kBT and contoured by color (low energy blue to high energy red). Distinctive local basins are well defined. The centroid of each contour is indicated, and the shift amounts can be measured in each direction. (B) Table of the shift amounts in each PC component due to mutation. These values do not appear to correlate well with the remainder of the scores.


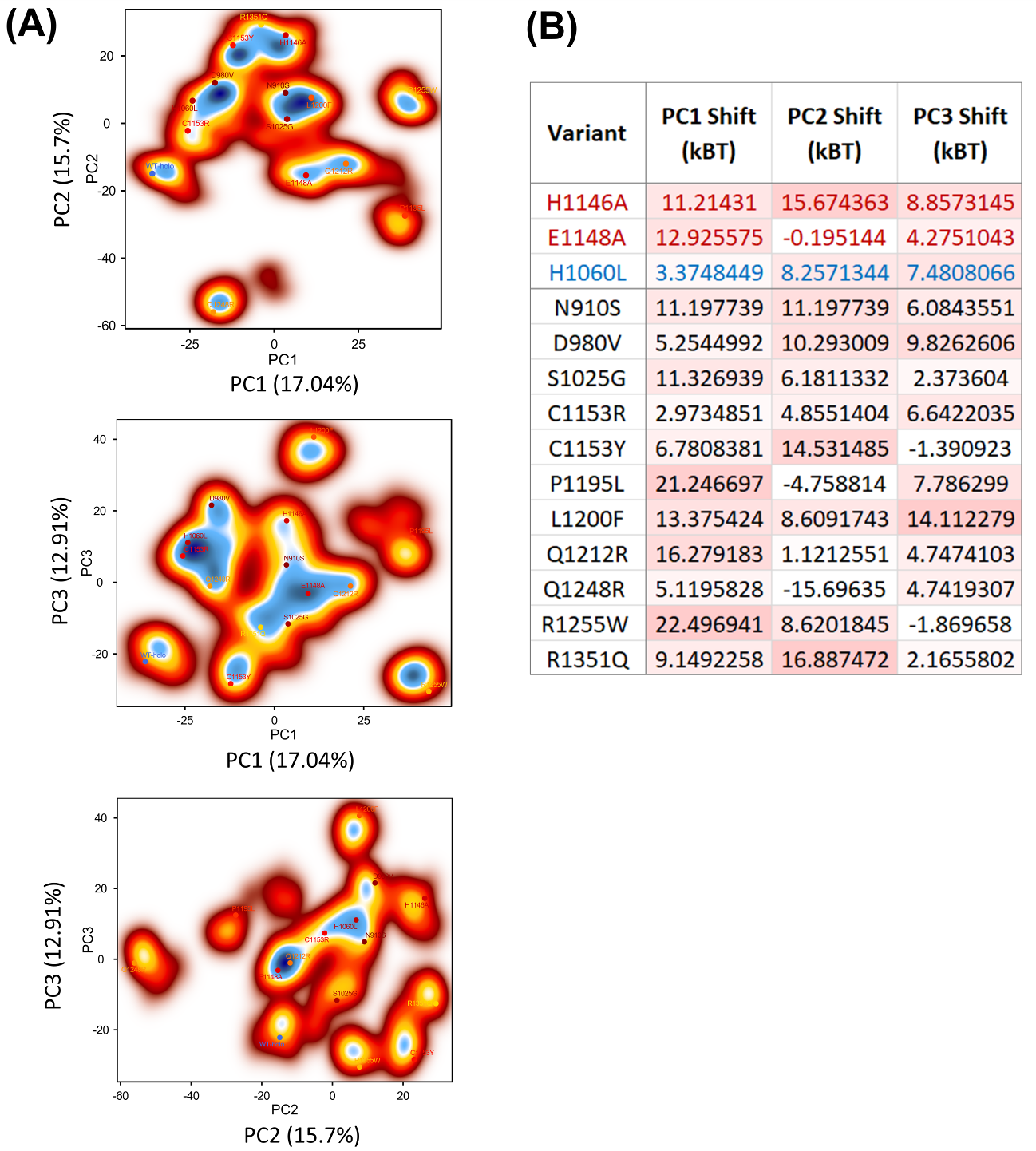


**Figure S6**

**Figure S6.** Sequence alignment of the KDM6 family proteins. Alignment of the amino acid sequences of human KDM6A/UTX, KDM6C/UTY, and KDM6B/JMJD3. Residue numbers are based on KDM6A. Secondary structure elements are shown at the top of each sequence alignment. Cyan (benign) and red (damaging) triangles at the bottom indicate the position of the control variants while orange triangles indicate the position of the Kabuki-associated variants.


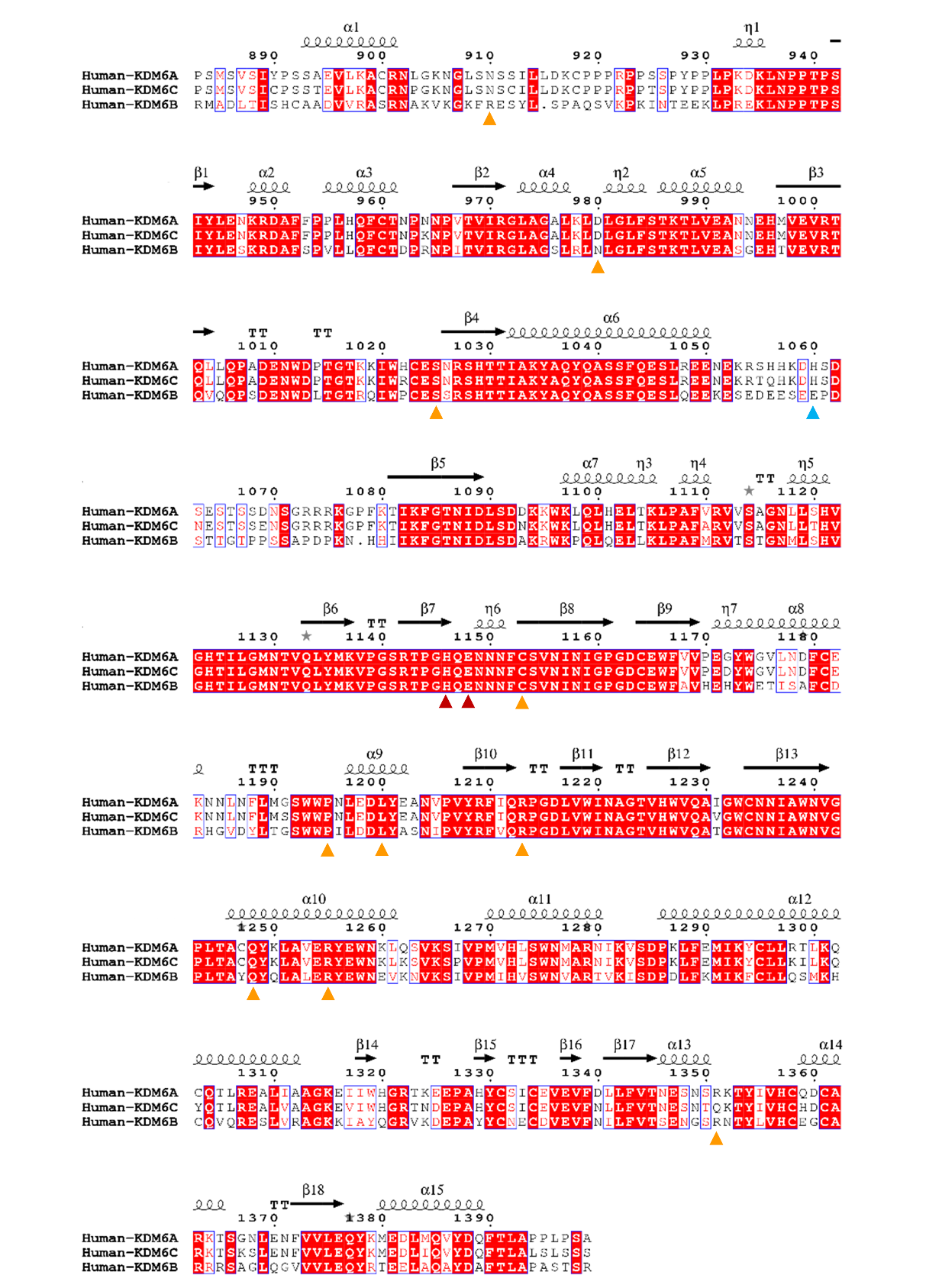


**Text S1. R studio codes used for generating the figures**

(1) pKa shift heatmap (Figure 3A)

library(readxl)

df<-read_excel("C:\\Users\\ychi\\Desktop\\pKa-Kabuki-syndrome.xlsx")

df

library(dplyr)

dat <- df[, 3 : ncol(df)] - df$WT

dat <- cbind(df$Residue, dat)

library(tidyr)

library(reshape)

dat_new <- melt(dat)

library(ggplot2)

#svg(filename="pKa-shift.svg", width=8, height=8, pointsize=9)

colnames(dat_new) <- c("Residue", "Variant", "pKa")

plot (p <- ggplot(dat_new, aes(Variant, Residue)) +geom_tile(aes(fill = pKa), colour = "white") + scale_fill_gradient2(low = "red", mid = "whitesmoke", high = "blue") + theme(axis.text.x = element_text(angle = -90, hjust = 0, vjust = 0.5, size = 12), axis.text.y = element_text(hjust = 0, vjust =0.5, size = 3)))

#dev.off()

(2) RMSD violin plot (Figure 3B)

library(readxl)

rmsd <- read_excel("C:\\Users\\ychi\\Desktop\\RMSD-Kabuki-variants-10replicates-last-500frames.xlsx")

rmsd

library(reshape)

rmsd_plt <- as.data.frame(t(rmsd))

rmsd_plt

write.csv(rmsd_plt, "C:\\Users\\ychi\\Desktop\\RMSD-mod.csv")

df <- read.csv("C:\\Users\\ychi\\Desktop\\RMSD-mod.csv", skip = 1)

df

df <- melt(df)

df

#pdf('rvETscore_viol.pdf', height = 5, width = 8)

library(ggplot2)

ggplot(df, aes(x=variable, y=value, fill=variable)) +

geom_violin(trim=T) +

geom_boxplot(width=0.1, fill="white", outlier.shape=NA) +

theme(plot.title = element_text(hjust = 0.5))+

labs(title="RMSD distribution") +

labs(y = "RMSD (Å)") +

labs(x = "Variants") +

scale_fill_manual(values = c("blue", "green", green", green", "red", "red", "orange", "orange", "orange", "orange", "orange", "orange", "orange", "orange", "orange", "orange", "orange", "orange", "orange")) +

scale_y_continuous(limits = c(0.7, 2.0)) +

theme(axis.text.x = element_text(angle = 45, vjust = 1, hjust=1), panel.background = element_rect(fill = "white"), axis.line = element_line(size = 0.5, linetype = "solid", colour = "black"))

#dev.off()

(3) The porcupine plot (Figure 2D) and the free energy landscape (Supplementary Figure S4A) of PC analysis were made using the in-house pipeline workflow (Dr. Michael T. Zimmermann), which will be published and available upon request. The remainder of the figures were made by using either PyMol or Microsoft Excel program.
